# Optimizing image registration and infarct definition in stroke research

**DOI:** 10.1002/acn3.388

**Published:** 2017-01-20

**Authors:** George W. J. Harston, David Minks, Fintan Sheerin, Stephen J. Payne, Michael Chappell, Peter Jezzard, Mark Jenkinson, James Kennedy

**Affiliations:** ^1^ Acute Vascular Imaging CentreRadcliffe Department of MedicineUniversity of OxfordLevel 2, John Radcliffe HospitalOxfordOX3 9DUUnited Kingdom; ^2^ Department of NeuroradiologyOxford University Hospitals NHS TrustJohn Radcliffe HospitalOxfordOX3 9DUUnited Kingdom; ^3^ Department of Engineering ScienceInstitute of Biomedical EngineeringUniversity of OxfordOld Road Campus Research BuildingOxfordOX3 7DQUnited Kingdom; ^4^ Oxford Centre for Functional MRI of the BrainNuffield Department of Clinical NeurosciencesUniversity of OxfordJohn Radcliffe HospitalLevel 6, West WingOxfordOX3 7DQUnited Kingdom

## Abstract

**Objective:**

Accurate representation of final infarct volume is essential for assessing the efficacy of stroke interventions in imaging‐based studies. This study defines the impact of image registration methods used at different timepoints following stroke, and the implications for infarct definition in stroke research.

**Methods:**

Patients presenting with acute ischemic stroke were imaged serially using magnetic resonance imaging. Infarct volume was defined manually using four metrics: 24‐h b1000 imaging; 1‐week and 1‐month T2‐weighted FLAIR; and automatically using predefined thresholds of ADC at 24 h. Infarct overlap statistics and volumes were compared across timepoints following both rigid body and nonlinear image registration to the presenting MRI. The effect of nonlinear registration on a hypothetical trial sample size was calculated.

**Results:**

Thirty‐seven patients were included. Nonlinear registration improved infarct overlap statistics and consistency of total infarct volumes across timepoints, and reduced infarct volumes by 4.0 mL (13.1%) and 7.1 mL (18.2%) at 24 h and 1 week, respectively, compared to rigid body registration. Infarct volume at 24 h, defined using a predetermined ADC threshold, was less sensitive to infarction than b1000 imaging. 1‐week T2‐weighted FLAIR imaging was the most accurate representation of final infarct volume. Nonlinear registration reduced hypothetical trial sample size, independent of infarct volume, by an average of 13%.

**Interpretation:**

Nonlinear image registration may offer the opportunity of improving the accuracy of infarct definition in serial imaging studies compared to rigid body registration, helping to overcome the challenges of anatomical distortions at subacute timepoints, and reducing sample size for imaging‐based clinical trials.

## Introduction

The Acute Stroke Imaging Research Roadmap (ASIRR) III outlines the future challenges for stroke imaging research following the remarkable series of positive endovascular stroke trials.^1^ In particular, it emphasizes the need for accurate definition of final infarct volume in imaging‐based early phase clinical trials, which potentially reduces sample size and accelerates the translation of novel treatments into clinical practice.^2^ However, the optimum method for defining final infarction is not clear: serial infarct volumes across timepoints correlate, but the measured volumes are different owing to the effects of incomplete infarction at early timepoints, and anatomical distortion such as edema and atrophy at later timepoints.^3^, ^4^, ^5^ The ASIRR II, a precursor to the ASIRR III, took a pragmatic approach defining an early timepoint (18–36 h) diffusion‐weighted image for standardized final infarct measurement, recognizing these confounds to infarct volume measurement, but also that imaging follow‐up may be limited by logistical issues and incomplete due to mortality.^6^

The importance of coregistration of multimodal imaging is emphasized in the ASIRR III.^1^ The post hoc analysis of the Echoplanar Imaging Thrombolytic Evaluation Trial (EPITHET) dataset unmasked a positive effect of alteplase following within timepoint coregistration of the perfusion‐ (PWI) and diffusion‐weighted (DWI) imaging.^7^, ^8^ Yet, while image coregistration using rigid body registration within timepoints is increasingly being used in clinical trials, for example, within the Rapid processing of PerfusIon and Diffusion (RAPID) software,^9^, ^10^ tracking infarction across timepoints is most commonly approached using nonregistered volumetric analysis.^11^

Nonlinear image registration, a complimentary approach to rigid body registration, is utilized as a processing step to enable the consistent analysis of large cohorts for methods such as functional imaging to accommodate intersubject differences in brain morphology. Nonlinear registration also offers the opportunity to track tissue outcome more accurately in the same subject where the structure of the brain varies across timepoints. When employed in a treatment study in neuro‐oncology, which is confronted by the same issues as stroke of evolving pathology and differing treatment responses, nonlinear registration of the pre and posttreatment scans revealed clinically relevant treatment effects.^12^

The hypothesis of this study was that nonlinear image registration would improve the consistency of infarct volume definition over rigid body registration in serial imaging over a month period following acute stroke, assessed by comparing absolute volumes and overlap metrics of infarction at different timepoints in the same patients. This was tested using manual and automated approaches in the definition of tissue outcome masks using both DWI‐ and T2‐weighted FLAIR imaging. Finally, the impact of the two different registration techniques on infarct definition and trial sample size calculation was explored.

## Methods

### Patients

Consecutive eligible patients with ischemic stroke were recruited into a prospective observational cohort study regardless of age or stroke severity under research protocols agreed by the UK National Research Ethics Service committees (references: 12/SC/0292 and 13/SC/0362). Inclusion criteria for this analysis were as follows: clinical diagnosis of stroke within 18 h of symptom onset; patient or representative able to give a clear medical history and participate in the consent process; age over 18. Patients with a contraindication to MRI, or severely impaired conscious level (score >1 on question 1a of the National Institute for Health Stroke Scale) were excluded. Any patient with recurrent stroke within the follow‐up period was excluded.

### Imaging protocols

Patients were imaged at presentation, 24‐h (18–48 h), 1‐week (3–9 days), and 1‐month (14–42 days) timepoints, whenever possible. All scans were acquired using a 3.0T Siemens Verio scanner (Siemens Healthcare, Erlangen, Germany). Scanning protocols included diffusion‐weighted imaging (3 directions, 1.8 × 1.8 × 2.0 mm, FoV = 240 mm, 4 averages, b = 0 and 1000 sec/mm^2^, repetition time = 9000 msec, TE = 98 msec, 50 slices, 2 min 53 sec) with apparent diffusion coefficient calculation and T1‐weighted structural imaging (MPRAGE, 1.8 × 1.8 × 1.0 mm, FoV = 228 mm, TR = 2040 msec, TE = 4.55 msec, 3 min 58 sec) at all timepoints; and T2‐weighted turbo spin echo FLAIR (1.9 × 1.9 × 2.0 mm, FoV = 240 mm, TR = 9000 msec, TE = 96 msec, TI = 2500 msec) at 1‐week and 1‐month timepoints. When intravenous thrombolysis was indicated the presenting MRI scan occurred during the alteplase infusion. For patients presenting beyond the time window for thrombolysis the presenting MRI was acquired as soon as possible after admission.

### Infarct definition

Manual infarct definition was independently performed by a neuroradiology fellow (DM) and a stroke fellow (GH) on the 24‐h diffusion‐weighted b1000 image (b = 1000sec/mm^2^), 1‐week and 1‐month T2‐weighted FLAIR image using the masking tool in FSLView.^13^ All infarct masks were reviewed and a consultant neuroradiologist (FS) resolved any discrepancies. Semiautomated delineation of ADC infarct volumes at 24 h was performed using the threshold of 620 × 10^6^ mm^2^/sec identified as optimal for defining infarct at presentation (for details see Data S1).^14^ Analysis was restricted to voxels within tissue masks created using the FSL Automated Segmentation Tool.^15^ Mirrored contralateral masks were created to facilitate control tissue analysis in standard (MNI152) image space.^16^

### Registration steps

Registration within each timepoint was to the respective T1‐weighted structural scan. The rigid body registration schedule (6 degrees of freedom, 3 rotations, and 3 translations) available in FMRIB's Linear Registration Tool (FLIRT) was used.^17^, ^18^

Registration across timepoints was either rigid body, using FLIRT as described above, or nonlinear, using FMRIB's nonlinear registration tool (FNIRT). Rigid body registration aligns two images from the same patient without deformation of image structure. In contrast, FNIRT generates a deformation warp that maps voxels of one structural image onto another according to relative signal intensity, and can correct for anatomical differences between images.^13^ The transformation matrix from FLIRT and warp‐field coefficients from FNIRT was generated by registering the structural T1‐weighted image at each timepoint to the presenting T1‐weighted image. The default settings for FNIRT were applied as this methodology is optimized for T1‐ to T1‐weighted registrations, with addition of input‐weighting masks to prevent registration errors due to infarct‐related T1‐weighted signal change at later timepoints.

### Statistics

All comparisons were made following registration to the presenting structural T1‐weighted image space, apart from the comparison of ADC with b1000 image masks at 24 h, which was made in the native diffusion image space. Following registration to the presenting structural image space, a threshold of 50% was applied to masks to minimize errors associated with blurring introduced by the transformation or deformation. In this way all infarct volumes were compared following their relevant registration step. Absolute and relative differences between infarct volumes generated by each registration technique were compared for each timepoint.

Agreement of infarct masks across and within timepoints by registration technique was evaluated using volume overlap statistics, including mean overlap and union overlap (Data S1.), and false‐positive rate (FPR).^19^, ^20^ Where all imaging timepoints were available, the overlap statistics of the infarct volume for each registration technique were generated to determine how consistently the infarcts registered to the presenting structural image. Any disparities in infarct definition at different timepoints were further explored through visual inspection of the images. CNRs and interrater agreements prior to discrepancy resolution were calculated for each method of infarct definition.

The 1‐week (T2‐weighted FLAIR) and the 24‐h (b1000 and ADC) infarct volumes were compared to the 1‐month T2‐weighted FLAIR infarct. The 1‐month timepoint was chosen as the reference image because infarction would be complete. The manual b1000 image masks and semiautomated ADC masks were compared to each other in native diffusion image space, and to follow‐up T2‐weighted FLAIR imaging after registration to the presenting structural scan.

Contrast‐to‐noise ratios (CNRs) of the infarct masks at each timepoint by imaging sequence were quantified (Data S1.). Interrater agreements following manual infarct definition were quantified using the mean overlap agreement.

In addition, the effect on sample size in a hypothetical clinical trial attributable to the two different registration techniques was calculated. The underlying assumptions were of an absolute minimum detectable difference in mean infarct volumes of 20% at the 0.05 two‐tailed significance level, with a power to demonstrate the null hypothesis of 0.8, using a two‐sample *t* test.^21^ Analysis of the effect of lesion size was explored by dividing patients into two groups of greater or less than median infarct volume. All statistical analyses were performed using Prism 6 (GraphPad Software, Inc., La Jolla, CA) and STATA 13 (StataCorp, College Station, TX).

## Results

### Patient details

Thirty‐seven patients met the criteria for inclusion (mean age: 72; female: 51%; median onset to presenting MRI: 3 h 25 min). All underwent MRI scanning at presentation, and 17 at every available timepoint. Patient demographics are presented in Table 1.

**Table 1:** Demographic data

Number of patients	37
Mean age (SD), years	72 (16)
Female sex, %	51
Thrombolysed, %	41
Prior stroke/TIA, %	24
Hypertension, %	49
Diabetes mellitus, %	16
Atrial fibrillation, %	30
Cigarette smoker (current), %	19
Median NIHSS (IQR)	7 (3−14)
Median ED to MRI (IQR), h:mm	1:38 (1:07−4:54)
Median onset to MRI (IQR), h:mm	3:25 (2:50−6:45)
Median lesion volume on MRI (ADC) at admission (IQR), mL	3.5 (0.5−11.6)

SD, standard deviation; TIA, transient ischemic attack; NIHSS, National Institute for Health stroke scale; ED, emergency department; MRI, magnetic resonance imaging; IQR, interquartile range; ADC, apparent diffusion coefficient.

### Rigid body versus nonlinear registration

**Figure 1:**
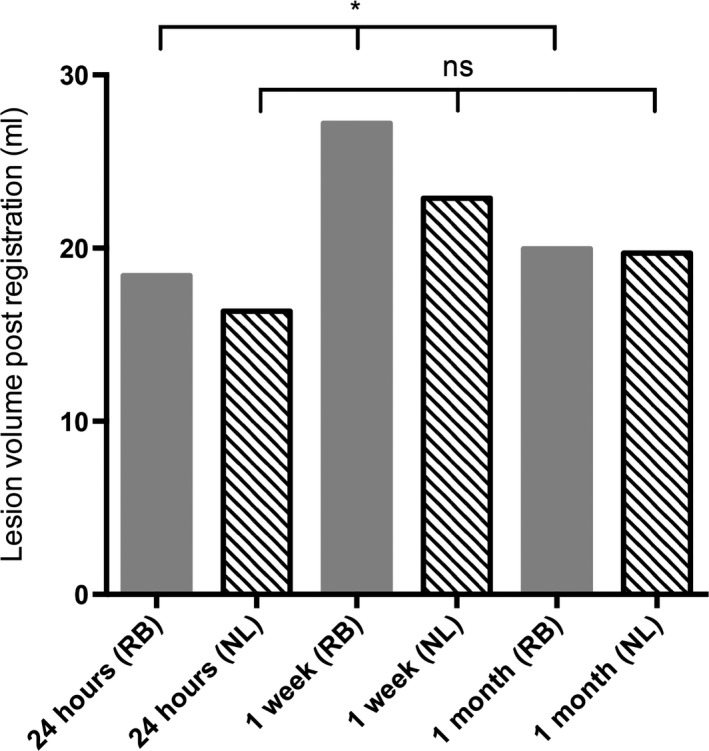
Mean infarct volumes following rigid body (RB, solid fill bars) or nonlinear (NL, hatched bars) registration to the presenting structural image where all three endpoints were available. 17 patients. ANOVA: *P < 0.05; ns, not significant.

Infarct volumes differed significantly across timepoints following rigid body registration to the presenting image space (Fig. 1 and Table 2). Mean infarct volumes were greatest at 1 week, followed by 1 month and 24 h when all imaging timepoints were available (ANOVA, *P* = 0.045). The difference in infarct volumes was less marked following nonlinear registration mean infarct volume (*P* = 0.058).

**Table 2:** Volume changes and overlap agreements of lesion masks following nonlinear registration to the presenting structural image compared to rigid body registration of the same masks

Time point	Number of patients	Volume following rigid body registration, mL	Volume following nonlinear registration, mL	Paired t‐test of volumes	Percentage change, %	Mean overlap, %	Union overlap, %
24 h	27	30.1	26.1	0.004	−13.1	90.6	82.9
1 week	30	39.0	31.9	0.04	−18.2	87.6	77.9
1 month	29	17.5	17.2	0.13	−2.2	91.1	83.7

Nonlinear registration led to significant reductions in mean infarct volumes at 24 h and 1 week, compared to rigid body registration, but not at 1 month (Table 2). The greatest change in mean infarct volumes occurred for the 1‐week scan, which resulted in 7.1 mL reduction, compared to 4.0 mL at 24 h. This was reflected in the reduction in individual infarct volumes following nonlinear registration both at 24 h (median change = −4.4%, interquartile range = −12% to −0.8%) and 1 week (−7.7%, −12.5% to −3.0%), but not at 1 month (−2.1%, −5.3% to +4.5%). Nonlinear registration also improved overlap agreements across timepoints.

**Figure 2:**
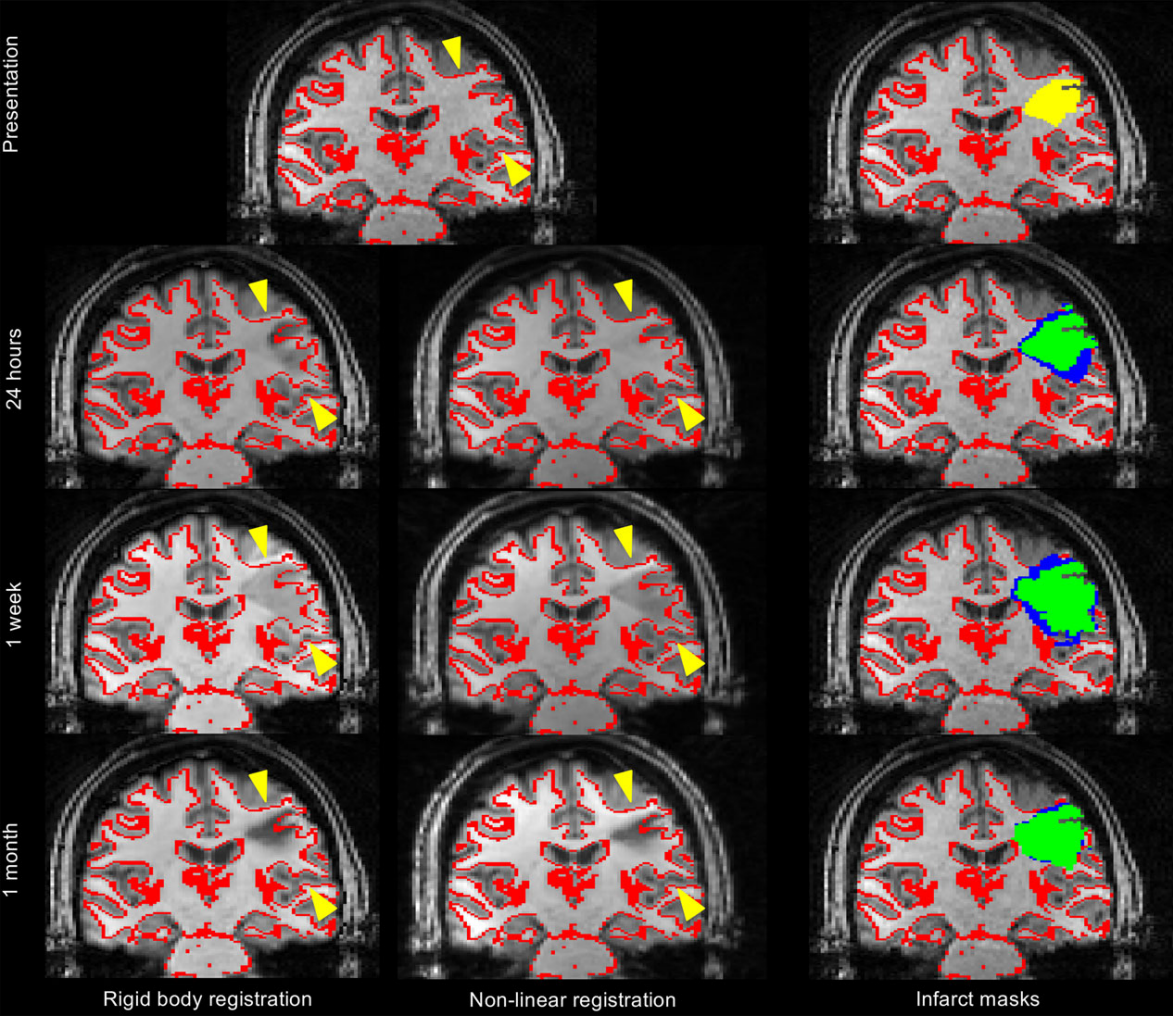
Rigid body versus nonlinear registration in a patient presenting with right‐sided weakness and sensory loss. The top row shows the presenting T1‐weighted structural image with the gray‐white matter interface (red) overlaid for reference, and with the presenting ADC infarct overlaid (yellow, top right image). The lower rows show the T1‐weighted structural images from each follow‐up timepoint (24 h, 1 week, and 1 month) registered to the presenting scan using either rigid body or nonlinear registration, with the presenting gray‐white matter interface overlaid for reference (red). Yellow arrows highlight the regions where rigid body registration does not correct for subacute edema at 24 h and 1 week. The right hand column shows the infarct masks from each time point overlaid on the presenting T1‐weighted structural image using either rigid body (blue) or nonlinear registration (green).

**Figure 3:**
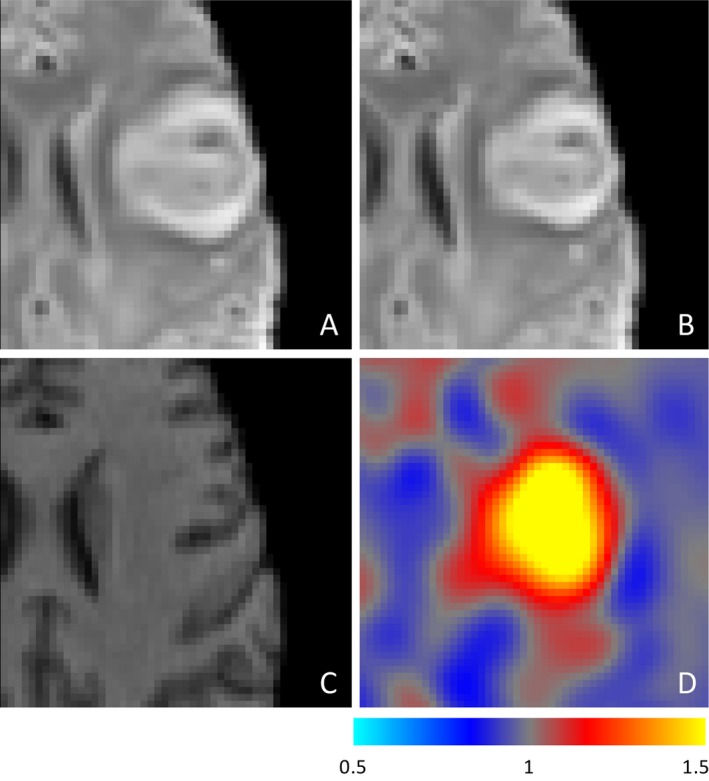
Example of the ability of nonlinear registration to correct for distortion due to edema and hemorrhagic transformation at 1 week. T2‐weighted FLAIR images registered using rigid body registration (A) and nonlinear registration (B) to the presenting T1‐weighted structural image (C). Panel D shows the Jacobian output of the nonlinear registration, where the intensity reflects the degree of compression required in each voxel, demonstrating that nonlinear registration is correcting for distortion associated with the stroke lesion itself rather than improving registration more generally.

Visual inspection of the imaging revealed that the volume changes and improved overlap agreements following nonlinear registration were as a result of correction for anatomical distortion at 1 day and 1‐week. An example is shown in Figure 2 secondary to hemorrhage and edema at 1‐week. The nonlinear registration of the T2‐weighted FLAIR image at 1 week accommodated the anatomical distortions that remained following rigid body registration. Maximal compression occurred within and close to the infarct as demonstrated on visualization of the Jacobian transformation (Fig. 3). Subgroup analysis of patients with and without known reperfusion showed similar trends in improved absolute volume and overlap agreement (Tables S2 and S3).

### Comparison of outcomes using nonlinear registration

**Figure 4:**
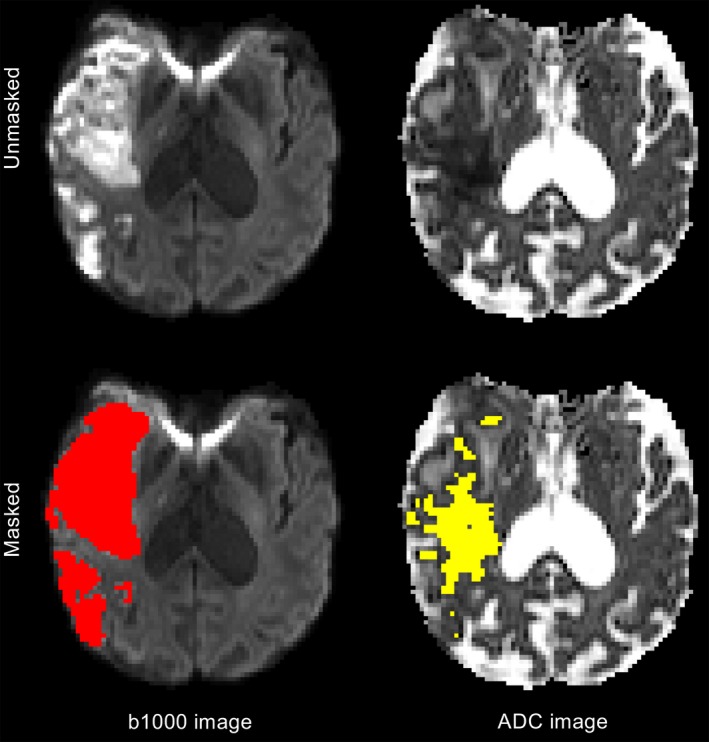
b1000 diffusion‐weighted (left) and apparent diffusion coefficient (ADC, right) images at 24 h from stroke onset. Manually delineated lesion mask of the b1000 image (red) is more sensitive to infarction than the automatically generated ADC lesion mask (yellow) as it excludes regions of diffusion pseudonormalization.

Using both volume agreement and overlap statistics the 1‐week infarct overestimated infarction compared to the 1‐month infarct (Fig. 1, 2, Table S1), regardless of registration method. Visual inspection of the imaging revealed that this apparent overestimation was driven by the 1‐month masks underestimating infarct volume due to a reduction in T2‐weighted signal intensity by 1‐month. Neither uncorrected edema at 1 week nor atrophy at the later time appeared to make a major contribution. Figure S1 highlights these issues using scans from a representative patient. In contrast, 24‐h diffusion imaging underestimated infarct volume at 1‐month with a sensitivity of 62% using b1000 image and 49% using ADC (Table S1). The direct comparison of b1000 and ADC infarct masks in diffusion image space confirmed the low sensitivity of ADC defined lesions due to the normalization of ADC values on diffusion imaging at 24 h (Fig. 4).

Prompted by the demonstration of the underestimation of infarct volume using the 1‐month scan, further analyses were performed using the 1‐week nonlinearly registered infarct mask as the reference image. The 24‐h b1000 image showed low sensitivity to the absolute infarct volume as defined on the 1‐week scan, with correspondingly modest overlap statistics (Fig. 1 and Table 3). Although the 1‐month infarct volumes were more similar to those at 1‐week than the 24‐h infarct volumes, this was accompanied by a higher false‐positive rate and not by improved sensitivity. Inspection of the infarct masks revealed that the higher false‐positive rate was typically attributable to the inclusion of white matter hyperintensity not linked to the index event and the lower contrast‐to‐noise ratio when defining lesions at 1 month (Data S2.).

**Table 3:** Comparison of optimized 24‐h and 1‐month lesion masks with 1‐week T2‐weighted FLAIR lesion masks. Between scan registration was nonlinear

Comparator	Number of patients	Mean difference in volume to 1‐week scan, mL (%)	Mean overlap, %	Union overlap, %	False‐positive rate, %	Sensitivity, %
24‐h b1000 image	22	−10.5 (−27.0)	77.4	63.1	8.0	66.9
1‐month FLAIR	24	−3.4 mL (−19.9)	74.9	59.8	17.7	68.6

### Sample size effects

In the setting of a hypothetical trial with sample size calculations derived from these data, nonlinear registration of images to the presenting images led to a reduction in sample size for the overall group of 13.2% when compared to the use of rigid body registration. This effect was consistent across both larger and smaller infarct volume§, with the reduction in sample size ranging from 7.3% to 15.5% (see Table 4).

**Table 4:** The impact of registration technique on sample size according to infarct volume (above or below median lesion volume) on 1‐week T2‐weighted FLAIR. SD, standard deviation

	Rigid body mean (±SD) lesion volume, mL	Nonlinear mean (±SD) lesion volume, mL	Sample size change by using nonlinear registration, %
Below Median	2.2 (±1.86)	2.1 (±1.71)	−7.3
Above Median	75.9 (±94.3)	61.8 (±70.7)	−15.5
Overall group	39.0 (±75.5)	31.9 (±57.7)	−13.2

## Discussion

This study demonstrates that nonlinear registration improves image alignment across timepoints compared to rigid body registration resulting in more consistent absolute volumes across timepoints and improved overlap statistics. Secondly, a b1000 image is more sensitive than using a consistent predetermined ADC threshold from presentation to 24 h when defining infarction using DWI at 18–36 h.^6^ T2‐weighted FLAIR imaging at 1‐week nonlinearly registered to the presenting image was the most sensitive method for defining final tissue infarction, not identified on either 24‐h b1000 imaging or 1‐month T2‐weighted FLAIR imaging. Finally, it highlights that the use of nonlinear registration in the image analysis of stroke clinical trials could lead to a significant reduction in sample size required.

Rigid body registration ensures that the representation of voxels is fixed relative to standardized brain landmarks. This static integration of images lends itself to the colocalization of pathology within timepoints as was employed in the positive post hoc analysis of Echoplanar Imaging Thrombolytic Evaluation Trial (EPITHET).^7^ A recent study demonstrated that comparing changes in absolute infarct volumes significantly underestimated lesion growth compared to estimates derived from linearly registered serial images.^22^ However, if the pathology is dynamic, as it is across timepoints following stroke with anatomical distortion due to infarct growth, edema, atrophy and hemorrhage, then those standardized landmarks may no longer be consistent and the underlying assumptions for rigid body registration are undermined.

Nonlinear registration was developed as a technique to accommodate the differences in brain morphology across a wide range of subjects by transforming the data into a standardized space to allow analysis to take place. This study demonstrates that by taking advantage of this feature, nonlinear registration can also be used to more accurately track dynamic tissue outcome within an individual, independent of reperfusion status. This is reflected in the improvement in consistency of infarct volumes and overlap agreement metrics across timepoints, and improved alignment of landmarks on visual inspection. Nonlinear registration may reduce clinical trial sample size, by reducing measurement error by an average of 7 mL at one week in this cohort. The effect on sample size in a hypothetical clinical trial was seen across all infarct volumes suggesting that it may be beneficial to use nonlinear registration in all trials with acute timepoints, regardless of whether the underlying stroke mechanism is lacunar or large vessel occlusion.

The ASIRR II recommends DWI at 24 h as the preferred imaging outcome for assessing final infarct volume.^6^ ADC maps allow the objective automated segmentation of infarction, an advantage that has been exploited by the Rapid processing of PerfusIon and Diffusion (RAPID) software to enable accurate point of care treatment decisions to be made.^8^, ^14^ However, this study demonstrates that using the same ADC threshold for defining infarct at 24 h as used at presentation leads to a significant underestimation of infarct volume at 24 h when compared with the b1000 image. While this study demonstrated the near identical threshold at presentation for defining infarction (615 vs. 620×10^6^ mm^2^/sec, using the same criteria), the major contribution to the low sensitivity to infarction at 24 h was ADC pseudonormalization.^23^, ^24^, ^25^, ^26^ b1000 imaging captures not only restricted diffusion, but also T2‐weighted hyperintensity or “shine‐through”,^25^ which is less susceptible to dynamics of ADC values. This makes manual definition of the b1000 image a more sensitive biomarker of infarction at 24 h than the semiautomated ADC definition, and suggests future development of automated segmentation tools to define infarct volumes should be focused on b1000 imaging.

While acknowledging the pragmatic concerns that led to ASIRR II to recommend a 24 h imaging outcome, this study demonstrates that the nonlinear registration of the 1‐week T2‐weighted FLAIR is the most accurate representation of final infarct volume of the three timepoints explored. Infarct volume continues to evolve in the days poststroke, and while the 24‐h b1000 image may closely correlate with final infarct, these volumes tend to underestimate final infarct volume.^4^, ^27^ Signal loss and contrast reduction, combined with partial volume effects at the infarct boundaries, contribute significantly to the inferiority of the 1‐month T2‐weighted FLAIR image.

### Limitations

While the improved overlap metrics and attendant volume changes would support nonlinear registration as an improvement over rigid body registration for across timepoint alignment, it is unlikely that all structural distortions have been corrected by the nonlinear registration. Any residual uncorrected image registration may introduce error, in addition to the blurring introduced by the transformation or deformation of the regions of interest. Spatial resolution and partial volume effects make further contributions, but these will likely be consistent and, therefore, unbiased.

Defining accuracy of image registration is challenging, because there is no “gold standard”.^28^ However, use of independently defined infarct volumes at each timepoint, and their improved consistency of volumes and overlap coefficients at multiple timepoints, together with observations such as those in Figure 2, highlight the advantages of nonlinear registration in this setting.

This study is limited by its small size, inclusion of less severe strokes, and the missed scans in some patients. Further work includes the need to validate these findings in a larger cohort of patients, which would allow the effect of reperfusion status and lesion size within appropriately sized subgroups to be explored. In addition, the impact of factors such as anatomical locations and different imaging sequences (for instance, the utility of nonlinear registration in serial CT imaging) would be valuable.

## Conclusions

Image registration is an overlooked but crucially important aspect of acute stroke clinical trial design. The adoption of automated rigid body image registration has been important for the purposes of stratification of patients at presentation into acute stroke clinical trials. This study shows that where tracking tissue outcome following acute stroke over time is required, nonlinear image registration improves confidence in the definition of infarction. In particular, nonlinear registration helps overcome the challenges of imaging distortion at subacute timepoints highlighted in the ASIRR III and offers the opportunity to improve clinical trial implementation by limiting lesion measurement error.

## Conflict of Interest

None declared.

## Supporting information


**Data S1**. Supplementary methods.
**Data S2.** Supplementary results.
**Figure S1.** T2‐weighted signal intensity on FLAIR images at 1 week (left) and 1 month (right) from the same patient, nonlinearly registered to the presenting scan.
**Table S1.** Comparison of 24‐h and 1‐week infarct masks with 1‐month T2‐weighted FLAIR infarct masks using nonlinear registration between timepoints.
**Table S2.** Volume changes and overlap agreements of lesion masks following non‐linear registration to the presenting structural image compared to rigid body registration of the same masks, divided into patients with known reperfusion or non‐reperfusion.
**Table S3.** Comparison of optimized 24‐hour and 1‐month lesion masks with 1‐week T2‐weighted FLAIR lesion masks, divided into patients with known reperfusion or non‐reperfusion. Between scan registration was non‐linear.
