# Supplementary material for: Optimizing image registration and infarct definition in stroke research
Source: Ann Clin Transl Neurol. 2017 Jan 20;4(3):166–74. doi: 10.1002/acn3.388 (PMC5338168; doi:10.1002/acn3.388)
Supplement: Supplementary file 1 — Data S1. Supplementary methods. Data S2. Supplementary results. Figure S1. T2‐weighted signal intensity on FLAIR images at 1 week (left) and 1 month (right) from the same patient, nonlinearly registered to the presenting scan. Table S1. Comparison of 24‐h and 1‐week infarct masks with 1‐month T2‐weighted FLAIR infarct masks using nonlinear registration between timepoints. Table S2. Volume changes and overlap agreements of lesion masks following non‐linear registration to the presenting structural image compared to rigid body registration of the same masks, divided into patients with known reperfusion or non‐reperfusion. Table S3. Comparison of optimized 24‐hour and 1‐month lesion masks with 1‐week T2‐weighted FLAIR lesion masks, divided into patients with known reperfusion or non‐reperfusion. Between scan registration was non‐linear. [file ACN3-4-166-s001.pdf]

Supplementary Methods

Automated infarct definition

The final ADC-defined infarct mask was generated following three steps. Cluster-based analysis eliminated smaller, noisy clusters. The largest cluster was identified and, in the second step, smoothed with a Gaussian kernel of standard deviation 1mm to remove thin connections between otherwise separate clusters.[1] Finally, the single largest remaining cluster was selected and re-masked by the original mask to eliminate boundary spreading due to the previous smoothing step. The resulting cluster volume was selected as the infarct mask. ADC masks were inspected by a clinician to ensure that the correct cluster had been selected, and if necessary reselected. Where more than one region was though to represent infarction, this cluster was added to the infarct mask.

Contrast to noise ratios (CNRs)

CNRs were derived using the following formula:

$$CNR = \frac{(\mu_l - \mu_c)}{\sigma_c}$$

Where μ_l is the mean intensity within the lesion mask, μ_c is the intensity in the contralateral region, and σ_c is the standard deviation of the signal in the contralateral lesion.

Overlap agreements

Overlap agreement of defined ROIs between and within time points was evaluated using volume overlap statistics. These were mean overlap (MO, also known as Dice

coefficient) and union overlap (UO, also known as Jaccard coefficient) measures. False positive rate (FPR), and sensitivity (also known as target overlap) were calculated.

$$MO = \frac{2\times(S \cap T)}{T + S}$$

$$UO = \frac{S \cap T}{S \cup T}$$

$$FPR = \frac{S \setminus T}{S}$$

$$Sensitivity = \frac{S \cap T}{T}$$

Where *S* is the comparator (or source) region being mapped onto the reference (or target) region, *T* (Figure).

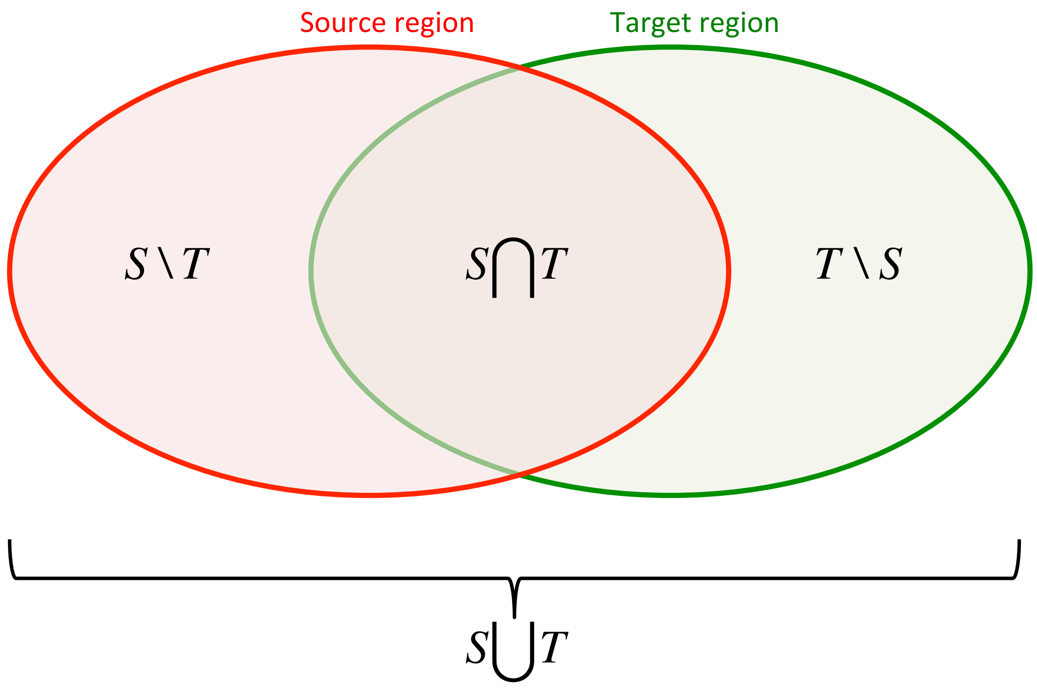

1. Smith SM, Brady JM. SUSAN - A new approach to low level image processing. Int J Comput Vision 1997;23:45-78.

Supplementary Results

Contrast-to-noise ratios and inter-rater agreements

The mean CNRs were lowest for 24-hour ADC infarcts (1.4). For the manually delineated infarcts, the highest mean CNR was the 24-hour b1000 image (7.0), followed by the 1-week T2-weighted FLAIR image (2.2), and the 1-month T2-weighted FLAIR image had the lowest CNR (1.9). The mean overlap agreement for manual infarct definition between the two raters prior to discrepancy resolution was 92%, 86% and 80% at 24 hours, 1 week and 1 month respectively. There was no significant correlation between infarct size and inter-rater overlap agreement at any timepoint.

Supplementary Figure

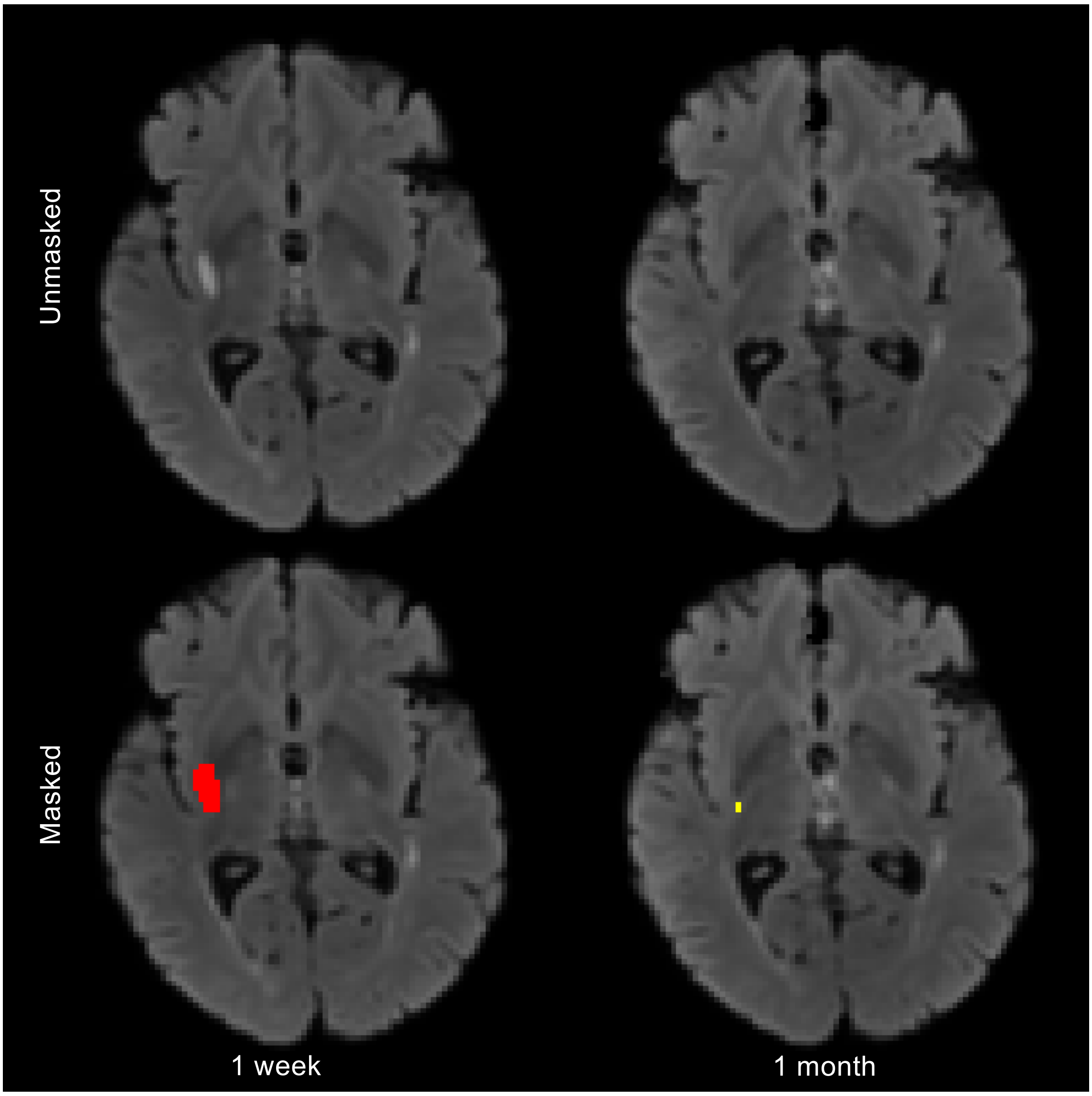

Supplementary Figure 1: T-2 weighted signal intensity on FLAIR images at 1 week (left) and 1 month (right) from the same patient, non-linearly registered to the presenting scan. The same images with the manually outlined masks superimposed are on the bottom row. Masks were outlined in native image space before being non-linearly registered to the presenting structural scan. T2-signal loss and partial volume effects result in a relative insensitivity to infarction at 1 month.

Supplementary Tables

Comparator	Number of patients	Mean difference in volume to 1-month scan, ml (%)	Mean overlap, %	Union overlap, %	False positive rate, %	Sensitivity, %
24-hour b1000 image	20	-3.8 (-18.0)	68.3	51.9	24.2	62.2
24-hour ADC	20	-8.8 (-39.8)	61.6	44.5	18.0	49.3
1-week FLAIR	24	3.4, (19.9)	74.9	59.8	31.4	82.3

Supplementary Table 1: Comparison of 24-hour and 1-week infarct masks with 1-month T2-weighted FLAIR infarct masks using non-linear registration between timepoints.

Time point	Reperfusion	Number of patients	Volume following rigid body registration, ml	Volume following non-linear registration, ml	Paired t-test of volumes	Percentage change, %	Mean overlap, %	Union overlap, %
24 hours	Y	7	15.9	12.8	0.03	-19.7	86.8	76.7
24 hours	N	6	75.3	67.2	0.06	-10.7	92.5	86.0
1 week	Y	6	28.8	23.8	0.03	-17.3	88.5	79.3
1 week	N	7	102.4	81.4	0.1	-20.6	87.0	76.9
1 month	Y	7	17.4	17.2	0.5	-1.2	88.5	79.4
1 month	N	6	42.9	42.8	0.8	-0.3%	92.5	86.1

Supplementary Table 2: Volume changes and overlap agreements of lesion masks following non-linear registration to the presenting structural image compared to rigid body registration of the same masks, divided into patients with known reperfusion or non-reperfusion.

Comparator	Reperfusion	Number of patients	Mean difference in volume to 1-week scan, ml (%)	Mean overlap, %	Union overlap, %	False positive rate, %	Sensitivity, %
24-hour b1000 image	Y	5	-5.4 (-28.4)	73.9	58.6	11.4	63.4
24-hour b1000 image	N	5	-24.9 (-23.9)	81.5	68.7	5.8	71.7
1-month FLAIR	Y	5	-8.9 (-47.9)	64.5	47.6	20.0	54.1
1-month FLAIR	N	6	-6.0 (-14.0)	80.2	67.0	14.2	75.3

Supplementary Table 3: Comparison of optimized 24-hour and 1-month lesion masks with 1-week T2-weighted FLAIR lesion masks, divided into patients with known reperfusion or non-reperfusion. Between scan registration was non-linear.
